# Effects of ultrasound-guided stellate ganglion block on intrapulmonary shunt and oxygenation in patients with single-lung ventilation

**DOI:** 10.3389/fsurg.2024.1438146

**Published:** 2024-12-23

**Authors:** Guoshao Zhu, Changsheng Su, Zhenming Kang, Jingyang Zeng, Shunyuan Li

**Affiliations:** Department of Anesthesiology, Quanzhou First Hospital Affiliated to Fujian Medical University, Quanzhou, Fujian, China

**Keywords:** ultrasound-guided stellate ganglion blockade, single-lung ventilation, intrapulmonary shunt, oxygenation, thoracoscopic pulmonary lobectomy

## Abstract

**Background:**

Single-lung ventilation (SLV) is a widely used procedure in thoracic surgery; however, it can lead to hypoxemia, which is attributed to intrapulmonary shunt and hypoxic pulmonary vasoconstriction. Stellate ganglion blockade (SGB) has shown protective effects during SLV in various pulmonary conditions. The objective of the study was to assess the clinical utility of ultrasound-guided SGB in patients undergoing thoracoscopic pulmonary lobectomy through a prospective clinical trial.

**Methods:**

This prospective randomized controlled double-blind trial included 116 patients who underwent SLV. After exclusion, 88 patients were randomly assigned to either the SGB group (*n* = 40) or control group (*n* = 39), with the latter receiving no SGB. Hemodynamics using oxygenation index (OI) and the pulmonary shunt fraction (Qs/Qt), respiratory mechanics using dynamic lung compliance (Cdyn) and mean airway pressure (P_mean_), and levels of pro-inflammatory factors (IL-6 and IL-8) were assessed as clinical outcomes after surgery.

**Results:**

SLV induced upregulation of P_mean_, Qs/Qt, and levels of IL-6 and IL-8 and downregulation of Cdyn and OI. Compared to the control group, the SGB group demonstrated significantly decreased P_mean_, Qs/Qt, IL-6, and IL-8 and increased Cdyn and OI, suggesting the protective effects of SGB in patients who received SLV.

**Conclusions:**

Ultrasound-guided SGB improves respiratory mechanics, hemodynamics, and inflammatory responses during SLV. Our findings suggest a protective role of SGB in reducing complications associated with SLV.

**Clinical Trial Registration:**

The study was registered in the Chinese Clinical Trial Registry (#ChiCTR2200063210).

## Introduction

Single-lung ventilation (SLV) is a widely adopted thoracoscopic technique shown to have promising efficacy in improving patient outcomes (1, 2). Despite this, hypoxemia resulting from poor oxygenation persists as a severe complication of SLV. Previous studies have shown that the collapse and loss of ventilation in the operated lung ultimately lead to a lack of oxygenation and blood return to the left heart. This imbalance of the ventilation/blood flow ratio and increased intrapulmonary shunt (3, 4) is associated with consequent hypoxemia and the initiation of lung self-regulation protection mechanisms, such as hypoxic pulmonary vasoconstriction (HPV) (5). Despite efforts to prevent the occurrence of intrapulmonary shunt and enhance arterial oxygenation during surgery, the incidence of intraoperative hypoxemia remains substantial, ranging from 9% to 27% (6, 7). Consequently, reducing intrapulmonary shunt and preventing hypoxemia during SLV remain critical needs in clinical practice.

The stellate ganglion, also known as the cervicothoracic ganglion, comprises sympathetic ganglia spanning from C3 to C7 and the initial thoracic sympathetic ganglion (8). Existing research concentrates on the protective role of stellate ganglion blockade (SGB) in the lungs—manifested through the inhibition of inflammatory responses, reduction of oxidative stress, suppression of cell apoptosis, regulation of pulmonary vascular tone, and maintenance of autonomic nervous system stability (9, 10). Noteworthy contributions by Liu et al. (11) highlighted the inhibition of pro-inflammatory factors TNF-a and IL-6 in severe trauma patients through SGB, thereby regulating early inflammatory reactions. In addition, SGB alleviates excessive peripheral vasoconstriction and rectifies abnormal hemodynamics by reinstating the balance between the sympathetic and vagus nerves (12). It is worth mentioning that unilateral SGB has been demonstrated to induce vasodilation in vessels innervated by postganglionic fibers, resulting in heightened blood flow velocity, increased blood flow, expanded vessel diameter, and a decrease in vascular resistance, thus theoretically impeding HPV (13). However, the effects of SGB on hypoxic pulmonary vasoconstriction and intrapulmonary shunt during SLV, as well as its potential to induce alterations in oxygen partial pressure, remain unclear.

Herein, this study aims to enroll patients undergoing thoracoscopic pulmonary lobectomy to employ ultrasound-guided SGB to observe its influence on intrapulmonary shunt and oxygenation during SLV, demonstrating the efficacy of this novel and practical approach to alleviate hypoxemia during SLV.

## Method

### Patients

Patients undergoing elective thoracoscopic pulmonary lobectomy at our hospital were intended to be included. The study was approved by Quanzhou First Hospital affiliated with Fujian Medical University. Informed consent was acquired from the patients. The study was registered in the Chinese Clinical Trial Registry (#ChiCTR2200063210).

### Inclusion and exclusion criteria

The inclusion criteria in this research were as follows: (1) patients scheduled for thoracoscopic pulmonary lobectomy, (2) age between 18 and 75 years, and (3) ASA classification I–III. Patients were excluded if they met the following conditions: (1) FEV1/FVC <70%, (2) history of asthma or COPD, (3) acute pulmonary infections, or previous pulmonary surgery (4) receiving atropine and continuous vasoactive drug infusion.

### Experimental grouping

The study was designed as a prospective randomized controlled double-blind trial, where participants were randomly assigned to either the SGB group or the control group. In the SGB group, the intervention involved ultrasound-guided right stellate ganglion blockade using 0.5% ropivacaine (7 ml) before anesthesia induction. The control group received standard anesthesia without SGB. Three anesthesiologists participated in the study: the first was responsible for pre-anesthetic stellate ganglion blockade and related preparations, the second for anesthesia induction and maintenance, and the third for clinical data collection and postoperative assessment. The latter two anesthesiologists were blinded to the group allocation.

### Stellate ganglion blockade

Upon entering the operating room, routine monitoring measures, including electrocardiogram (ECG), heart rate (HR), blood pressure (BP), and pulse oximetry (SpO2) should be taken by connecting the monitor. Before the induction of general anesthesia, ultrasound-guided SGB was performed on the SGB group. The patients were positioned supine, and under ultrasound guidance, the needle was advanced from the anterior aspect of the neck to the superior aspect of the longus colli muscle at the level of the transverse process of C7.

### Anesthesia protocol

Induction involved intravenous administration of fentanyl 0.4 μg/kg, propofol 1.5–2 mg/kg, and rocuronium 0.2 mg/kg. After endotracheal intubation, mechanical ventilation was initiated with maintenance of end-tidal carbon dioxide partial pressure between 35 and 45 mmHg. Anesthesia maintenance included continuous infusion of propofol and remifentanil to keep the BIS between 40 and 60. Fentanyl (5 μg) was administered 20 min before the end of surgery.

### Risk analysis

The following lists items used for risk analysis: (1) hemodynamic management involved interventions for deviations from baseline values: when intraoperative mean arterial pressure (MAP) increased by >20% of the basal value or heart rate (HR) exceeded 100 beats/min, intravenous sufentanil 0.1 μg/kg was administered, along with a deepening of anesthesia. In cases of inadequate response, uradil 12.5 mg was intravenously injected. Conversely, if MAP decreased by >20% from the basal value, anesthesia depth was adjusted, and infusion rates were increased. Ephedrine hydrochloride (5–10 mg) was administered if needed. Continuous vasoactive drug infusion was initiated if hypotension persisted, leading to the exclusion of the subject from the study. (2) Bradycardia (<45 beats/minute) prompted observation for surgical-related factors to evaluate whether surgical operation pulls the viscera, with readiness to suspend surgical stimulation at any time. (3) Anesthesia depth was assessed and adjusted. (4) If correction remained insufficient, atropine (0.5 mg IV) was administered, with repeat doses as needed, resulting in exclusion from the study. (5) Intraoperative complications such as massive hemorrhage or anaphylactic shock, particularly those related to SGB, were promptly addressed through emergency resuscitation and subsequent exclusion from the study. (6) Intermediate open surgery, if required, led to the exclusion of the study subject. (7) Failure of ultrasound-guided stellate ganglion block resulted in the exclusion of the subject from the study.

### Statistical analysis

Comparative analyses between the control group and SGB group were conducted using independent *t*-test or Mann–Whitney test for continuous variables and chi-square tests or Fisher's exact test for categorical variables. Continuous variables collected at multiple time points, including hemodynamic parameters, pulmonary dynamics, oxygenation indices, and inflammatory factors, underwent two-way ANOVA analysis with a Bonferroni *post hoc* test to assess within-group and between-group variations over time. The significance level was set at *p* < 0.05.

## Results

### Overview of patient recruitment and group assignment

**Figure 1:**
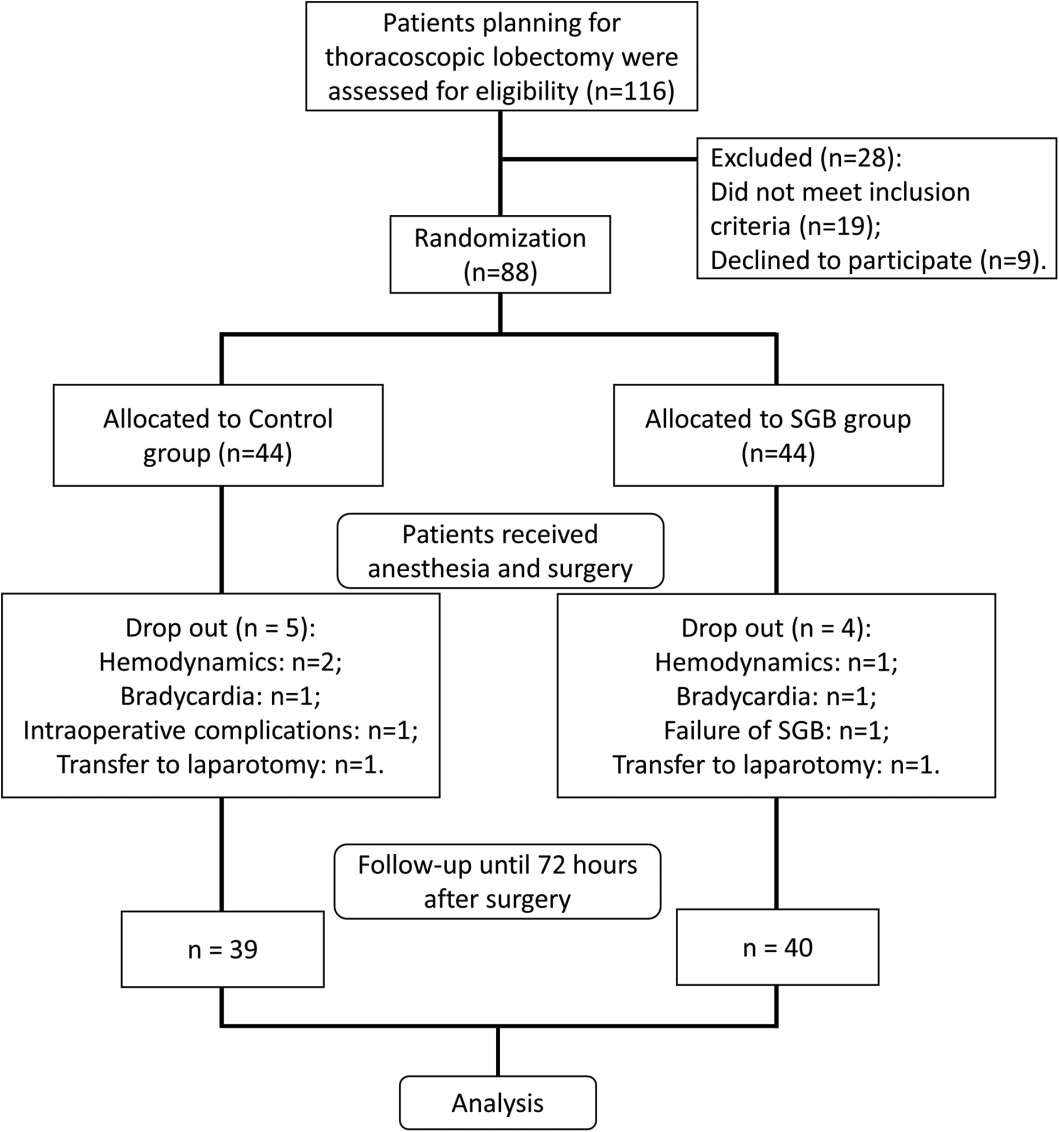
Study flow.

We evaluated 116 patients undergoing elective thoracoscopic pulmonary lobectomy at our hospital, with 19 patients not meeting the inclusion criteria and 9 refusing to participate (Figure 1). The eligible 88 patients were randomly divided into the SGB group and the control group. Each group received respective interventions. In the control group, two cases were excluded during surgery due to hemodynamic reasons, one case due to bradycardia, one case of intraoperative complications, and one case converted to open chest surgery. Finally, 39 cases were included in the analysis. In the SGB group, one case was excluded due to hemodynamic reasons, one case due to bradycardia, one case of SGB failure, and one case converted to open chest surgery. In the end, 40 cases were included in the analysis. In addition, comparing the baseline data of the two groups, there were no differences in terms of gender, age, surgical side, ASA classification, surgery duration, SLV, and pharmaceutical interventions (additional sufentanil use, uradil use, and ephedrine hydrochloride use) (Table 1).

**Table 1:** Demographic and clinical characteristics of the study participants.

Characteristics	Study group		*p*
	SGB (*n* = 40)	Control (*n* = 39)	
Gender			
Male	23 (57.5%)	19 (48.7%)	0.502
Female	17 (42.5%)	20 (51.3%)	
Age (years)	56.54 ± 10.16	55.91 ± 9.92	0.408
BMI (kg/m^2^)	22.71 ± 4.84	22.36 ± 4.79	0.372
Operative side			
Right	28 (70%)	25 (64.1%)	0.637
Left	12 (30%)	14 (35.9%)	
ASA scale			
I	8 (20%)	10 (25.6%)	0.822
II	18 (45%)	17 (43.6%)	
III	14 (35%)	12 (30.8%)	
Operation time (min)	179.28 ± 39.92	171.01 ± 41.27	0.247
OLV time (min)	135.78 ± 29.68	140.53 ± 28.89	0.196
Additional sufentanil use			
Yes	3 (7.5%)	4 (10.3%)	0.712
No	37 (92.5%)	35 (89.7%)	
Uradil use			
Yes	1 (2.5%)	1 (2.6%)	0.999
No	39 (97.5%)	38 (97.4%)	
Ephedrine hydrochloride use			
Yes	1 (2.5%)	2 (5.1%)	0.615
No	39 (97.5%)	37 (94.9%)	

Values were expressed as *n* (percentage, %) or mean ± SD. *p* values for each group were derived from either unpaired *t*-test or Mann–Whitney test as appropriate. Chi-square test or Fisher's exact test was used for assessing distribution of observations or phenomena between different groups.

BMI, body mass index; OLV, one-lung ventilation.

### Evaluation of respiratory mechanics

**Figure 2:**
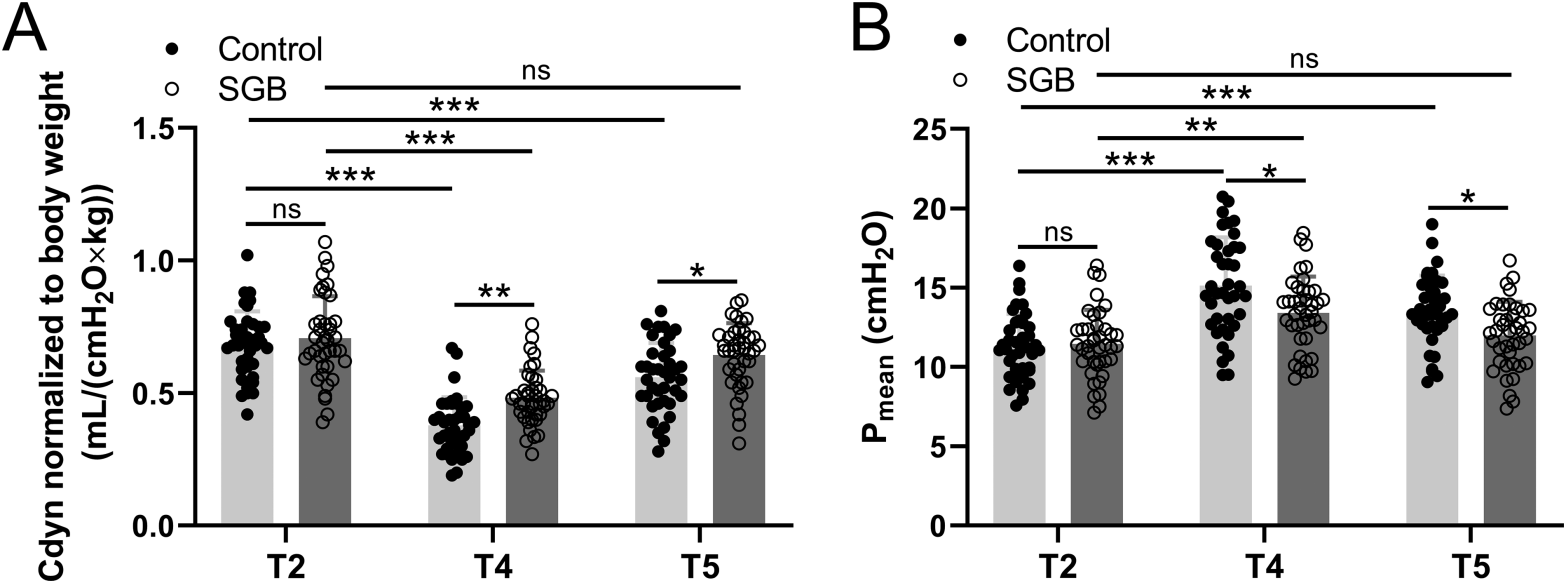
Comparisons of dynamic lung compliance (Cdyn) normalized to body weight **(A)** and mean airway pressure (P_mean_) **(B)** between the two groups at the time before single-lung ventilation (T2), 1 h since single-lung ventilation (T4), and 30 min after extubation (T5). Data were presented as mean ± SD showing all the data points. *n* = 40 in the SGB group and *n* = 39 in the control group. *p < 0.05, **p < 0.01, ***p < 0.001 and ns means no significance.

Hemodynamic parameters were further analyzed through the mean arterial pressure and heart rate of the two groups of patients at five time points: before induction of anesthesia (T1), before single-lung ventilation (T2), 30 min of single-lung ventilation (T3), 1 h of single-lung ventilation (T4), and 30 min after extubation (T5). Our data showed that the patients of the two groups had a decrease in blood pressure and heart rate during anesthesia and single-lung ventilation, and then recovered later on, but there was no significant difference between the two groups at each time point (Table 2). To evaluate how SGB affects respiratory mechanics, we employed the measurement of dynamic lung compliance (Cdyn, Figure 2A) and mean airway pressure (P_mean_, Figure 2B). In the control group, with the prolongation of time from T2 to T4 and T5, Cdyn significantly decreased at T4 (*p* < 0.001), while P_mean_ significantly increased at T4 (*p* < 0.0001) and T5 (*p* < 0.001). We further compared the changes in Cdyn and P_mean_ values of groups control and SGB group at T2, T4, and T5 time points, respectively. The SGB group caused a significant increase of Cdyn at T4 (*p* < 0.01) and T5 (*p* < 0.05) compared with the control group; however, a significant decrease in P_mean_ at T4 (*p* < 0.05) and T5 (*p* < 0.05). To sum up, SLV leads to a decrease in dynamic lung compliance and an increase in mean airway pressure, and the treatment of SGB weakens its impact.

**Table 2:** Comparison of MAP and HR between the two groups.

	MAP (mmHg)		HR (min)	
	SGB (*n* = 40)	Control (*n* = 39)	SGB (*n* = 40)	Control (*n* = 39)
T1	99.02 ± 11.14	98.54 ± 10.52	74.63 ± 6.81	73.94 ± 7.17
T2	89.16 ± 10.43	88.68 ± 10.18	68.44 ± 7.15	69.05 ± 7.38
T3	86.71 ± 8.92	85.57 ± 9.74	65.18 ± 7.09	64.87 ± 7.22
T4	82.94 ± 9.26	82.69 ± 9.05	63.62 ± 6.88	63.01 ± 6.94
T5	94.37 ± 9.61	93.64 ± 9.16	69.92 ± 7.36	69.42 ± 7.21

Values were expressed as mean ± SD. No significance was observed between the two groups at the same time.

MAP, mean artery pressure; HR, heart rate.

### Evaluation of hemodynamics

**Figure 3:**
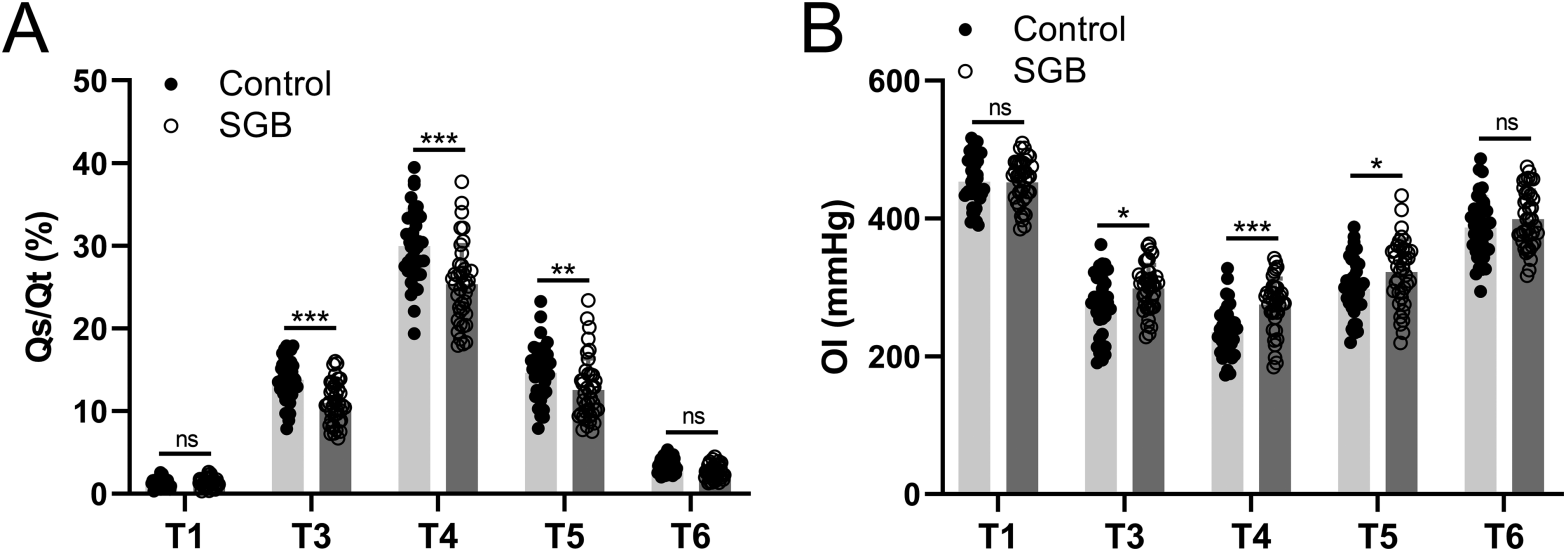
Comparisons of the intrapulmonary shunt (Qs/Qt, **A**) and oxygenation index (OI, **B**) between the two groups at the time before anesthetic induction (T1), 30 min since single-lung ventilation (T3), 1 h since single-lung ventilation (T4), 30 min after extubation (T5), and 24 h after surgery (T6). $\begin{array}{r} \mathrm{Qs}/\mathrm{Qt}\;=\;\left(\mathrm{Cc}O_{2}-\mathrm{Ca}O_{2}\right)/\left(\mathrm{Cc}O_{2}-\mathrm{Cv}O_{2}\right)\;\times \;100;\;\mathrm{Cc}O_{2}\;=\;1.34\;\times \;\mathrm{Hb}\;\times \;\mathrm{Sa}O_{2}\;+\;\left(713-\mathrm{PaC}O_{2}/0.8\right)\;\times \;0.0031 \end{array}$. OI = PaO_2_/FiO_2_. Data were presented as mean ± SD showing all the data points. *n* = 40 in the SGB group and *n* = 39 in the control group. *p < 0.05, **p < 0.01, ***p < 0.001 and ns means no significance.

We also assessed the changes in hemodynamics brought about by SGB. The pulmonary shunt fraction (Qs/Qt) and oxygenation index (OI) were monitored, and arterial blood and central venous blood were sampled at five time points (T1, T2–T6) for blood gas analysis to calculate the intrapulmonary shunt rate. In the control group, we measured Qs/Qt, and it reached a peak at T4 then decreased in subsequent measurements and at 24 h postoperatively (T6) almost returned to the same level as T1. However, the SGB group had significantly lower Qs/Qt values at T3 (*p* < 0.001), T4 (*p* < 0.001), and T5 (*p* < 0.01) compared to the control group (Figure 3A). Furtherly, from T3 to T6, OI value showed a tendency to decrease compared to T1, whereas the SGB group significantly increased OI at T3 (*p* < 0.05), T4 (*p* < 0.001), and T5 (*p* < 0.05) compared to the control group (Figure 3B). Our results suggested that SGB reversed the elevated Qs/Qt and reduced OI caused by SLV.

### Evaluation of inflammatory response

**Figure 4:**
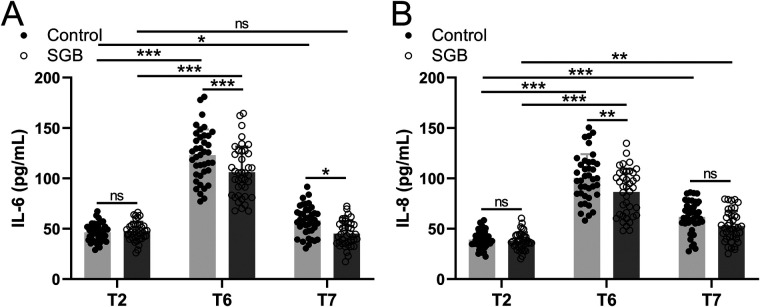
Comparisons of serum IL-6 **(A)** and IL-8 **(B)** between the two groups at the time before single-lung ventilation (T2), 24 h after surgery (T6), and 72 h after surgery (T7). Data were presented as mean ± SD showing all the data points. *n* = 40 in the SGB group and *n* = 39 in the control group. *p < 0.05, **p < 0.01, ***p < 0.001 and ns means no significance.

The SLV-induced inflammatory response was measured by the expression of inflammatory factors IL-6 and IL-8 in patients' peripheral blood at T2, T6, and 72 h postoperatively (T7) in Figure 4A. A significant upregulation of IL-6 was observed at T6 (*p* < 0.001) and T7 (*p* < 0.05) compared to T2 in the control group, but after treatment with SGB, it significantly downregulated at T6 (*p* < 0.001) and T7 (*p* < 0.05) comparing to the control group. Similar results were observed from the expression level of IL-8, i.e., significant upregulations at T6 (*p* < 0.001) and T7 (*p* < 0.001) compared to T2 in the control group, but after applying SGB, a significant downregulation of IL-8 was detected at T6 (*p* < 0.01) compared to the control group (Figure 4B). The expressions of IL-6 and IL-8 in this study indicate that SLV-related upregulations were suppressed by SGB.

## Discussion

This study aimed to address a critical clinical gap related to hypoxemia during SLV in patients undergoing thoracoscopic pulmonary lobectomy. Despite the widespread adoption of SLV in thoracic surgery, hypoxemia remains a significant complication, contributing to increased morbidity and mortality (14). HPV is a self-regulatory protective mechanism in the hypoxic state of the pulmonary circulation. HPV begins immediately after SLV treatment, after which it rises as the partial pressure of alveolar oxygen (PO2) decreases, and it has been shown that general anesthetics affect HPV, indicating choosing the right type of anesthesia intraoperatively is critical to the success of SLV (15). Furthermore, the mechanism that leads to hypoxemia is because of the persistent challenge of managing intrapulmonary shunt and optimizing oxygenation during SLV (16), implying an urgent need for alternative approaches to enhance oxygenation and mitigate complications during SLV (17, 18).

Limitations of previous studies include the suboptimal efficacy of existing interventions in addressing the multifaceted nature of hypoxemia during SLV, and efforts have been devoted to improving instruments such as optimizing ventilators and exploring different ventilation modes. The stellate ganglion, renowned for its ability to regulate inflammatory responses and maintain the stability of the autonomic nervous system, posed a distinctive target for intervention. SGB offered a novel intervention for improving respiratory and hemodynamic parameters during SLV (19), and the protective effects of SGB in various pulmonary conditions have been shown, making it a promising avenue for exploring its applications in SLV (20, 21). Moreover, the foundation of this study lay in the potential vasodilatory effects of SGB, which could result in heightened blood flow and reduced vascular resistance in innervated vessels (22). But to our best knowledge, we first observed improvement in dynamic lung compliance and reduction in mean airway pressure in the SGB group during SLV may indicate a potential benefit of SGB in reducing the impact of ventilation on pulmonary function.

Our results also detected the effect of SGB on increasing oxygenation and reducing intrapulmonary shunt, unlike anesthesia methods such as isoflurane (4). It is also worth noting that during SLV, ischemia, and reperfusion of lung tissue, repeated atrophic re-expansion and exposure to stimuli such as surgical operation and excessive stretching of lung tissues are applied, resulting in the promotion of the release of lung-derived inflammatory substances and aggravation of systemic inflammatory response. In this study, SGB also took a part in pulmonary protection through the downregulation of IL-6 and IL-8, which were induced by employing SLV.

These findings may pave the way for a novel, targeted intervention that addresses the complexities of hypoxemia during SLV, ultimately improving patient outcomes in thoracic surgery. The demonstrated improvements in pulmonary dynamics and inflammatory response in our study warrant further exploration in larger cohorts and varied clinical scenarios. Future studies should focus on patient stratification, refine the timing of interventions, and explore the long-term implications of SGB in thoracic surgery. While the study did not reveal a decisive impact on these parameters, the small variations observed may be indicative of a potential role for SGB in specific subsets of patients or under certain conditions. Additionally, the selected time points for data collection may not capture the full spectrum of changes induced by SGB during and after surgery. Our study supports the utilization of SGB in various diseases that involve SLV (23–26).

## Conclusions

Ultrasound-guided SGB could be a favorable intervention for mitigating SLV-induced alterations in HPV, intrapulmonary shunt dynamics, oxygen partial pressure, and inflammation.

## Data Availability

The original contributions presented in the study are included in the article/Supplementary Material, further inquiries can be directed to the corresponding author.
